# Comparison of the Effect of Enteral Feeding through the Bolus and Continuous Methods on Serum Phosphorus and Glucose Levels in Patients with Mechanical Ventilation: A Randomized Clinical Trial

**DOI:** 10.1155/2020/6428418

**Published:** 2020-09-08

**Authors:** Javad Seyyedi, Zahra Rooddehghan, Mostafa Mohammadi, Shima Haghani

**Affiliations:** ^1^Department of Medical Surgical Nursing, School of Nursing and Midwifery, Tehran University of Medical Sciences, Tehran, Iran; ^2^School of Nursing and Midwifery, Tehran University of Medical Sciences, Tehran, Iran; ^3^Imam Khomeini Hospital, Tehran University of Medical Sciences, Tehran, Iran; ^4^MSc in Biostatistics Nursing Care Research Center, Iran University of Medical Sciences, Tehran, Iran

## Abstract

**Introduction:**

Patients who are under mechanical ventilation in intensive care units need to have nutritional support. Also, feeding methods affect serum phosphorus and glucose levels, which are very important in weaning patients off the ventilator. Thus, this study is to compare the effects of both bolus and continuous enteral feeding methods on serum phosphorus and glucose levels in patients with mechanical ventilation.

**Methods:**

In this clinical trial study, 34 patients in the intensive care unit of Imam Khomeini Hospital affiliated to the Tehran University of Medical Sciences satisfied inclusion criteria and were randomly divided into control and intervention groups. Sampling was done between October and February 2018. The intervention group received continuous enteral feeding for one week, and the control group received nutrition by the bolus method. The blood glucose level was measured every six hours, and the serum phosphorus level was recorded at the beginning and the end of the intervention, based on the data entry form with respect to all ethical considerations. Data analysis was done by SPSS-20 software.

**Results:**

The serum phosphorus level was significantly increased in the intervention group (*P*=0.004) and in the control group (*P* < 0.001) and was compared with the previous intervention. No significant difference was found between the intervention and control groups before and after the intervention (*P*=0.22) and also one week after the intervention (*P*=0.14). There was also no significant difference between the glucose levels from day 1 to day 7 in the control group (*P*=0.33) and the intervention group (*P*=0.086). *Discussion*. Nutritional support in both bolus and continuous methods increased the serum phosphorus level. It indicates the importance of the nutritional method in controlling the phosphorus level in critically ill patients. However, there was no difference between the effects of dietary methods on blood glucose control.

## 1. Introduction

Intensive care unit is one of the most important sections in any hospital, where patients with acute and critical conditions receive care and treatment [1]. One of the important groups of the patients admitted to intensive care units is the ones with respiratory disorders who require artificial airway and mechanical ventilation for survival. Respiratory disorders include postoperative respiratory failure, head injury and trauma, stroke, rib fracture, chronic pulmonary obstruction associated with respiratory failure such as asthma and emphysema, and general illnesses such as myasthenia gravis, Guillen–Barre syndrome, and tetanus [2–4]. Descriptive findings show that patients who have been mechanically ventilated for a long time are nutritionally weak [5] and that gradually during their treatment, they develop hypercatabolism and increased metabolism due to physiological conditions and psychosocial stressors that are caused by their acute illness. Subsequently, patients will become malnourished if nutritional support is not sufficient to provide for body's demands [6]. Nutritional support in these patients is either enteral nutrition or total parenteral nutrition [7]. Enteral nutrition is mainly performed in two methods of continuous feeding over a period of 16–24 hours and bolus feeding for 4–6 times per day [8]. In the continuous method, feeding starts through a feeding pump at 20–50 cc/h and continues 24 hours a day. This method is more common in patients with respiratory failure as it reduces the risk of aspiration in these patients [9]. This method also provides a greater opportunity for absorption of micronutrients than other methods [10]. In the bolus method, nutrient (formula) is given to patients 4–6 times a day through a 50 mL syringe over a short interval of 4 –10 minutes [9]. This method is inexpensive and resembles the people's normal eating pattern. It also shortens the feeding time and is a good choice for patients with good digestive function, but the risk of aspiration is higher in this method than others due to delayed gastric emptying [10].

Nutritional methods also influence laboratory parameters [11–13]. Hypophosphatemia, which is defined as *P* ≤ 2.5 mg/dL [14], is one of the most common electrolyte abnormalities in ICUs, with 80% prevalence in the ICU patients. In fact, malnutrition with catecholamines, insulin, diuretics, alkalosis, diabetic ketoacidosis, and sepsis can predispose patients with acute conditions to hypophosphatemia [15, 16]. Hypophosphatemia can occur in ICU patients by three different mechanisms, including decreased intestinal absorption, increased renal excretion of phosphate, and redistribution of inorganic phosphate [17, 18]. Impairment of cellular energy storage as well as tissue hypoxia can lead to various clinical manifestations of hypophosphatemia [19], including myocardial dysfunction, diaphragm weakening, convulsions, coma, rhabdomyolysis, and RBCs' dysfunction [16]. Hypophosphatemia lowers the 2-3 levels of diphosphoglycerate in red blood cells, which shifts the oxyhemoglobin dissociation curve to left and increases hemoglobin tendency towards oxygen and subsequently reduces oxygen delivery to the tissues [20]. Studies have shown that hypophosphatemia disrupts weaning patients off the ventilator [21] and has been reported as one of the complications of intravenous nutrition [22]. However, changes in hypophosphatemia during continuous intestinal feeding have not been studied yet.

Hyperglycemia is another common disorder in ICUs, which is common even in nondiabetic patients [23]. Complications such as pneumonia, urinary tract infection, and surgical site infection are also associated with increased blood sugar [24–26]. Studies have shown that the blood glucose level affects the process of weaning patients off the ventilator and may be an indicator of the poor metabolic status in critically ill patients. It is also an important factor in determining the necessity for ventilation and disconnection the patient from the ventilator [11]. Compared with the intubated patients, the patients who have been successfully weaned off the ventilator have lower mean glucose concentration [11]. Studies have revealed that the speed and volume of feeding exert a direct impact on the blood glucose management [27]. In the study of Shahriari et al. that mostly concentrated on nonintubated patients, the mean blood glucose level was recognized to be lower in the patients with continuous feeding [28]. Given these assumptions and considering the importance of glucose and phosphate in weaning patients off the ventilator, the selection of a correct nutritional method is an important factor in caring the patients under mechanical ventilation. Thus, we decided to conduct a study with the aim of examining the effects of both continuous and bolus feeding methods on the phosphate and blood glucose levels in patients being treated under mechanical ventilation.

## 2. Materials and Methods

This study is a clinical trial that was conducted on two groups of intervention and control following the code of ethics: IR.TUMS.FNM.REC. Sampling has been done between October 2018 and February 2018 in the intensive care unit of one of the teaching hospitals of the Tehran University of Medical Sciences. The data were collected in two stages: before and one week after the intervention. Inclusion criteria were restricted to the patients aged between 18 and 85 years, who did not suffer from any gastrointestinal problems such as diarrhea, ileus, ostomy, obstruction, or chronic renal failure. They also did not have any history of intestinal feeding. The case study patients were admitted to the ICU while they had been intubated in the unit. We have also received the patients' families' consent to be included in the study. Exclusion criteria included the patient's death, any changes in feeding method, and feeding intolerance or termination.

**Figure 1:**
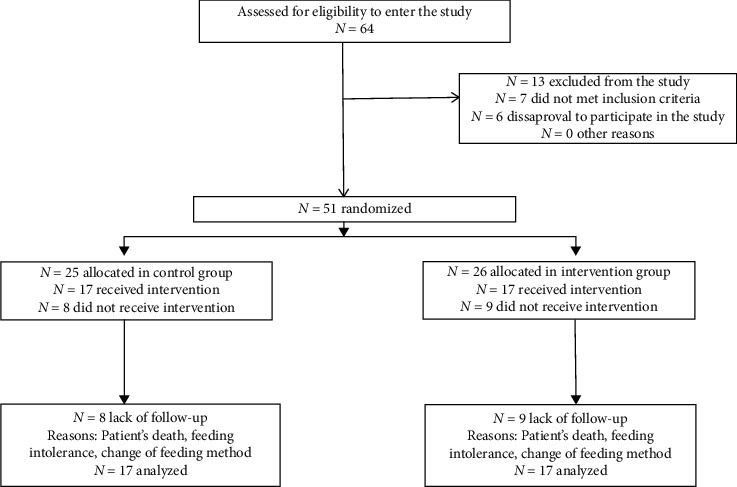
Flow diagram of the study.

Of 64 patients eligible for the study, 13 were excluded from the study (6 due to family disapproval, 2 due to gastrointestinal surgery, 2 due to diarrhea at baseline, and 3 due to chronic renal failure). Informed consent was obtained from the family of patients who met the inclusion criteria, and then, the patients were randomly divided into two groups of intervention and control according to the hospital number. The data collection methods were observation and measurement. The data collection tools included the patient record checklist and the data measurement checklist. The validity of the data collection tools was confirmed by 10 faculty members, and in order to ensure their reliability, the fixed glucometer kit and serum phosphorus measurement were used. To collect data in both groups, first baseline information such as age, sex, marital status, admission records, disease records, medications used, BMI, diagnosed disease, and level of consciousness were obtained from the patients' record and then were recorded in the baseline record sheet. At the beginning of the intervention, the serum phosphorus level was measured by the researcher using para-clinical tests. The blood sample kit and measurements were recorded in the laboratory data sheet. Then, feeding began in the intervention group by the continuous feeding method at 25 cc/h and in the control group by the routine bolus method via a nasal gastric (NG) tube at 75 cc/3 h with the commercial standard solution “Entera meal,” which contained 1 kcal of energy per cc (cubic centimeter). The patients were at semi-seated position during feeding time. On the first day of the patients' feeding, 50% of the patients' nutritional goal was met, and the volume of bolus was gradually increased every hour during the next 48 hours to achieve the ultimate nutritional goal (25 kcal/kg/day). This diet was kept until the end of study. During the sampling process, patients' blood glucose was measured every 6 hours using a CLEVER-CHEK glucometer and recorded in the patients' data sheet. According to Figure 1, in total, 17 participants left the study due to various reasons (7 due to death, 5 due to intolerance, 3 due to stop feeding, and 2 due to the change in feeding method).

Sampling continued until the desired sample size (34 individuals) was determined based on the study of Shahriari et al. [28] and the following formula:

(1) $$\begin{array}{c} n=\frac{{\left(z_{1-\left(\alpha /2\right)}+z_{1-\beta }\right)}^{2}\times \left(\sigma_{1}^{2}+\sigma_{2}^{2}\right)}{d^{2}}. \end{array}$$

When sampling was completed, baseline information such as age, sex, marital status, admission records, disease records, medications used, and diagnosed disease were analyzed using the SPSS software, version 20, and the hypothesis was tested by paired and independent *t*-tests.

## 3. Results

After the intervention, baseline data related to 34 patients were statistically analyzed using the Chi-square test, Fisher's test, and independent *t*-tests. According to Table 1, the mean age of patients was 62.12 years in the intervention group and 64.41 years in the control group. The two groups were homogenous in terms of age (*P*=0.67) and gender (*P*=0.49). Also, there was no significant relationship between variables such as history of hospital admission, history of disease and diagnosed disease, medication used, body mass index, and level of consciousness between the two groups.

As shown in Table 2, the results of the paired *t*-tests revealed that after intervention, the amount of phosphate in the control group (*P* < 0.001) and in the intervention group (*P*=0.004) were significantly different from the previous intervention. Before the intervention, the amount of phosphate in the control group was lower than that in the intervention group, and in this regard, there was no significant difference between the two groups before and after the intervention. However, the amount of phosphate in both intervention and control groups was significantly increased after the intervention. The results of the independent *t*-tests also showed no significant difference between the amount of phosphate in the intervention and control groups before the intervention (*P*=0.22) and one week after the intervention (*P*=0.14).

According to Table 3, the results of ANOVA tests related to repeated data showed no statistically significant difference in the glucose level from day 1 to day 7 in the control (*P*=0.33) and intervention (*P*=0.86) groups.

Results showed that the glucose level in the intervention group was lower than the control group, but the results of the independent *t*-tests showed no difference between the two groups in terms of glucose level during days 2 to 7 of the intervention (*P* > 0.05) except for day 1 (*P*=0.02).

## 4. Discussion

The importance of phosphorus, especially the role that it plays in the formation of adenosine triphosphate and 3-2 diphosphoglycerate, is related to the point that phosphorus is a very important component of supportive nutrition for patients [29]. Refeeding syndrome is one of the important issues in the feeding of malnourished patients. In these patients, insulin is released after feeding and the absorption of carbohydrates, which leads to the transfer of phosphate, magnesium, and potassium into the cell and thereby lowering the serum level of these electrolytes [30]. Studies also confirm this process [31–38]. Phosphorus reduction is a major indicator of this syndrome [39], which is defined as refeeding hypophosphatemia. This disorder is most severe in ICU patients at two intervals. The first interval is the first 12 hours of hospitalization where the patient has not received any nutrition. The second interval is 3–5 days after the start of the patients' artificial feeding [39, 40]. Hypophosphatemia has also been reported as one of the side effects of intravenous feeding [22], as patients who receive intravenous nutrition without phosphorus supplement develop severe hypophosphatemia [29]. However, the results of studies by Zeki et al. [41] revealed that the incidence of hypophosphatemia was higher in patients who had been fed through an NG tube compared with the patients who had received total parenteral nutrition. In the study of Agostino et al. [42], no significant difference was found between the phosphorus levels of the children being fed through an NG tube and children who received bolus nutrition. However, a study by Coşkun et al. [43] showed that all three groups of the patients who received intravenous, continuous enteral, and mixed-method nutrition developed hypophosphatemia, and also there was no significant relationship between the type of feeding and hypophosphatemia. Also, hypomagnesemia and hypokalemia were higher in the patients with hypophosphatemia. According to our data, 52.94% (*n* = 9) patients in the control group and 17.64% (*n* = 3) patients in the intervention group had electrolyte disorder as hypophosphatemia (*P* ≤ 2.5) before the start of intervention, which is consistent with the results of previous studies. However, the serum level of phosphorus increased significantly one week after the intervention in both intervention and control groups compared with preintervention time, which is in contradiction with the study results of Ramazan et al. This difference may be due to the type of the diet (Entera meal) used in the present study to feed patients, while the Nutrica formula was used in the study of Ramazan et al. to feed the patients.

In the other part of study, the mean blood glucose level in the intervention group increased with the increasing bolus volume from the second day, and then, with leveling bolus volume, the mean blood glucose also remained level. In the control group, in the second day, despite the increasing bolus volume, the mean blood glucose decreased compared with the intervention group and then remained level, but the mean and range of blood glucose level varied more than that in the intervention group. Studies have shown that the speed and volume of the patients' feeding have a direct impact on the management of blood sugar [44], and receiving large amounts of food at a specific time (similar to that of the bolus diet) increases the blood glucose level. This is while in the continuous feeding, the blood sugar level is better controlled [27]. In the present study, although the mean blood glucose level in the intervention group was lower, there was no significant relationship between the two groups in this regard.

## 5. Conclusions

According to the findings of the present research in both enteral bolus and continuous feeding methods, the level of serum phosphorus, which is an important factor in weaning patients off the mechanical ventilator [21], increased significantly, and in contrast with other studies, no significant difference in the mean blood glucose level between the two groups was recognized.

## Figures and Tables

**Table 1:** Comparison of baseline information between intervention and control groups.

Gender		Diabetes		Insulin received		Phosphate received		BMI	Age	Group
Male	Female	Yes	No	Yes	No	Yes	No			
7 (41.2%)	10 (58.8%)	3 (17.6%)	14 (82.4%)	3 (17.6%)	14 (82.4%)	2 (11.8%)	15 (88.2%)	23.51	62.12	Intervention
9 (52.9%)	8 (47.1%)	6 (35.3%)	11 (64.7%)	6 (35.3%)	11 (64.7%)	0 (0%)	17 (100%)	24.14	64.41	Control
*P*=0.49		*P*=0.43		*P*=0.43		*P*=0.48		*P*=0.44	*P*=0.67	

**Table 2:** Comparison of phosphate levels in the intervention and control groups one hour before and one week after the intervention, 2018.

Group	One hour before the intervention			One week after the intervention			Paired *t*-test
	Mean	SD	Maximum Minimum	Mean	SD	Maximum Minimum	
Control	2.62	1.20	−4.91 0.80	4.02	1.11	2.50 6	*t* = −4.98 d*f* = 16 *P* < 0.001
Intervention	3.04	0.66	−4.50 1.90	3.52	0.79	2 –4.50	*t* = −3.31 d*f* = 16 *P*=0.004
Independent *t*-test	*t* = −1.25 d*f* = 32 *P*=0.22			*t* = −1.48 d*f* = 32 *P*=0.14			

**Table 3:** Comparison of the glucose level in the intervention and control groups every 6 hours, and in the bolus and continuous feeding groups during one week of intervention, 2018.

Group	One hour before the intervention			One week after the intervention			Paired *t*-test
Glucose level	Mean	SD	Maximum Minimum	Mean	SD	Maximum Minimum	
Day 1	174.61	58.10	89.50 −315	134.44	36.34	91.50 −203	*t* = −2.41 d*f* = 32 *P*=0.02
Day 2	166.29	62.98	79.25 −350.50	142.27	4.59	97 −225	*t* = −1.32 d*f* = 32 *P*=0.19
Day 3	163.42	54.74	85.50 −318	145.95	4.52	95.50 −227.25	*t* = −1.05 d*f* = 32 *P*=0.29
Day 4	167.80	54.85	79.50 −298.50	141.8	43.30	95.50 −239.25	*t* = −1.57 d*f* = 32 *P*=0.12
Day 5	162.20	58.67	81.50 −302.50	144.77	44.35	96.25 −247.25	*t* = 0.97 d*f* = 32 *P*=0.33
Day 6	159	53.17	84.75 −300.50	144.39	39.46	108 −230	*t* = 0.90 d*f* = 32 *P*=0.37
Day 7	163.85	51.76	102.2 −307.25	141.64	39.87	104 −241.25	*t* = 1.40 d*f* = 32 *P*=0.17
ANOVA test of repeated data	*F* = 1.15 *P*=0.33			*F* = 1.91 *P*=0.086			

## Data Availability

The study will be published as an article, and the original data will not be shared with others.
